# Supplementary material for: Circumferential actomyosin bundles anchored by CCM1 drive endothelial cell contraction and vessel constriction
Source: Nat Commun. 2025 Dec 27;17:1056. doi: 10.1038/s41467-025-67820-3 (PMC12848307; doi:10.1038/s41467-025-67820-3)
Supplement: Supplementary file 1 — Supplementary Information [file 41467_2025_67820_MOESM1_ESM.pdf]

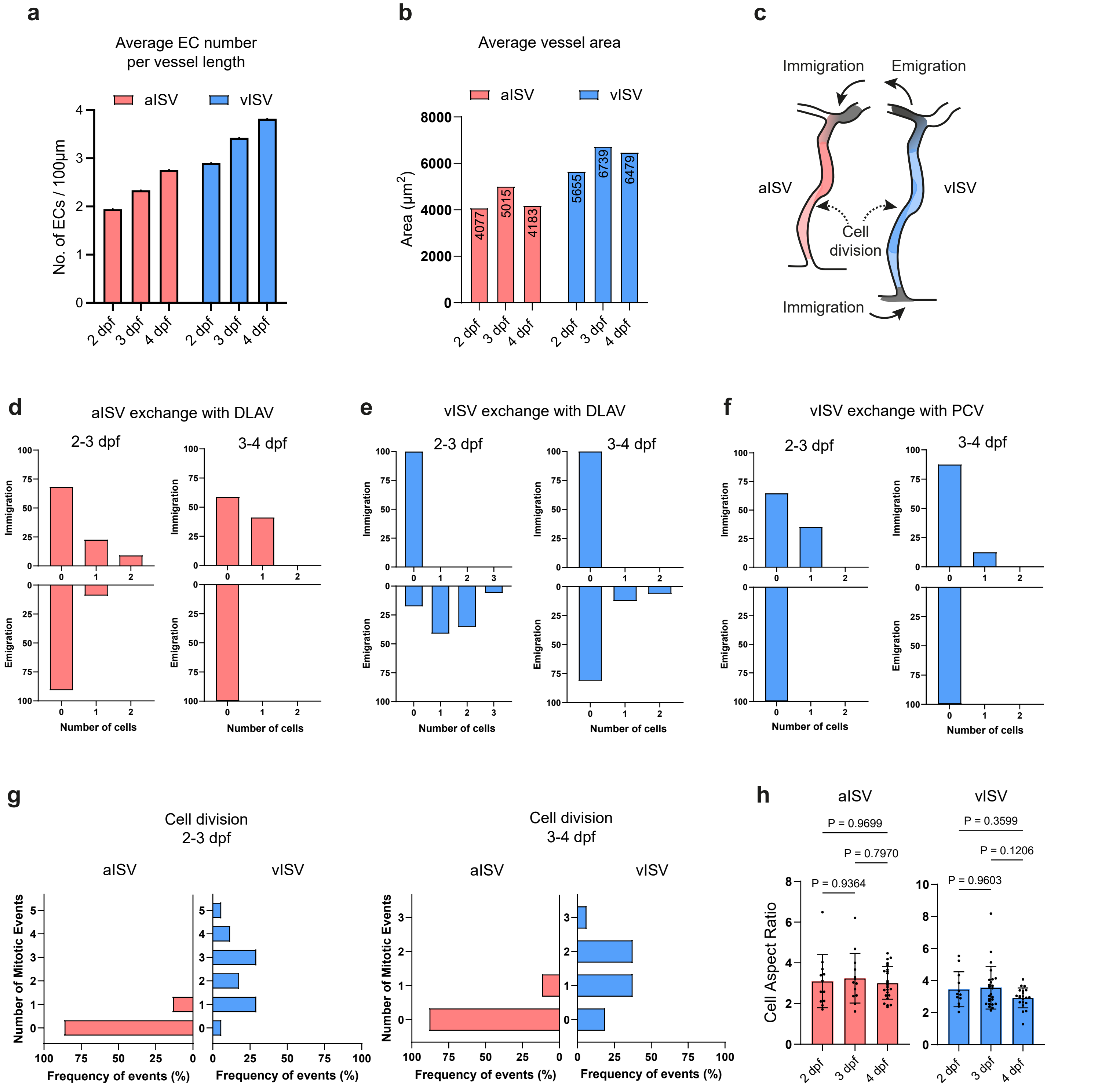

Supplementary Figure 1 Quantification of endothelial cell number, vessel area, and cellular behaviours during ISV remodelling.
a Average EC number per 100 µm of vessel length in aISVs and vISVs from 2-4 dpf (sample size of EC number and vessel length as in Fig. 1c-d).
b Average vessel area, calculated as π × average diameter × average length, in aISVs and vISVs from 2-4 dpf (sample size of EC number and vessel length as in Fig. 1b, d).
c Schematic illustrating cell immigration, emigration, and division in aISVs and vISVs.
d-f Quantification of cell immigration and emigration events. d aISVs exchange with DLAV; e vISVs exchange with DLAV; f vISVs exchange with PCV. Frequency of events (affected ISVs) and number of cells exchanged are shown.
g Number and frequency of aISVs and vISVs showing cell division events at 2-3 and 3-4 dpf (sample size of observed vessels as in Fig. 1e-f).
h EC aspect ratio in aISVs and vISVs from 2-4 dpf (sample size of cells as in Fig. 2b).
Statistical significance was assessed by one-way ANOVA with Tukey's multiple comparisons test. Data are shown as mean ± SD.
DA, dorsal aorta; DLAV, dorsal longitudinal anastomotic vessel; PCV, posterior cardinal vein; ISV, intersegmental vessel; aISV, arterial ISV; vISV, venous ISV.

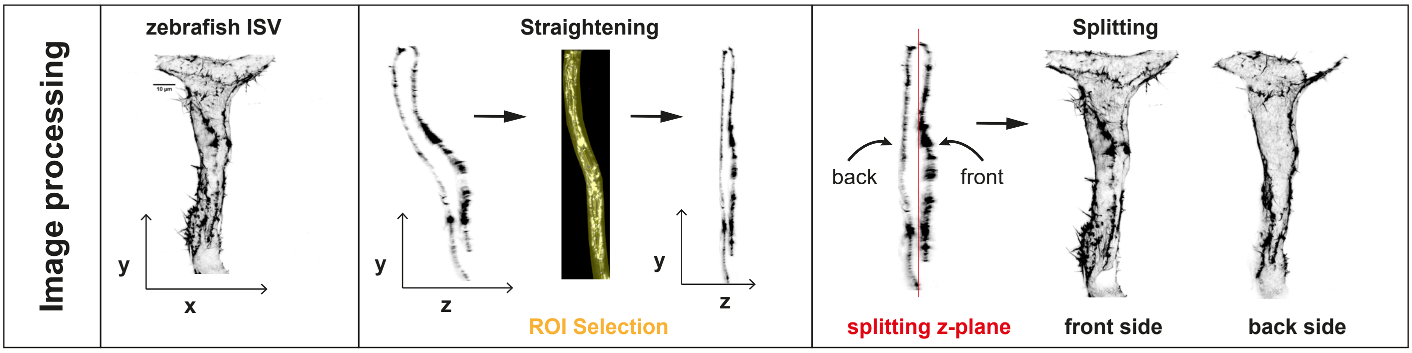

Supplementary Figure 2 Workflow for separating front and back sides of intersegmental vessels.
Illustration shows image processing workflow for separating front and back sides of ISVs. Image z-stacks of ISVs were straightened in Fiji, split into front and back halves, and projected by maximum intensity projection. A custom Fiji macro semi-automated straightening and splitting, with manual input for spline tracing (yellow ROI) and z-plane selection (red line).

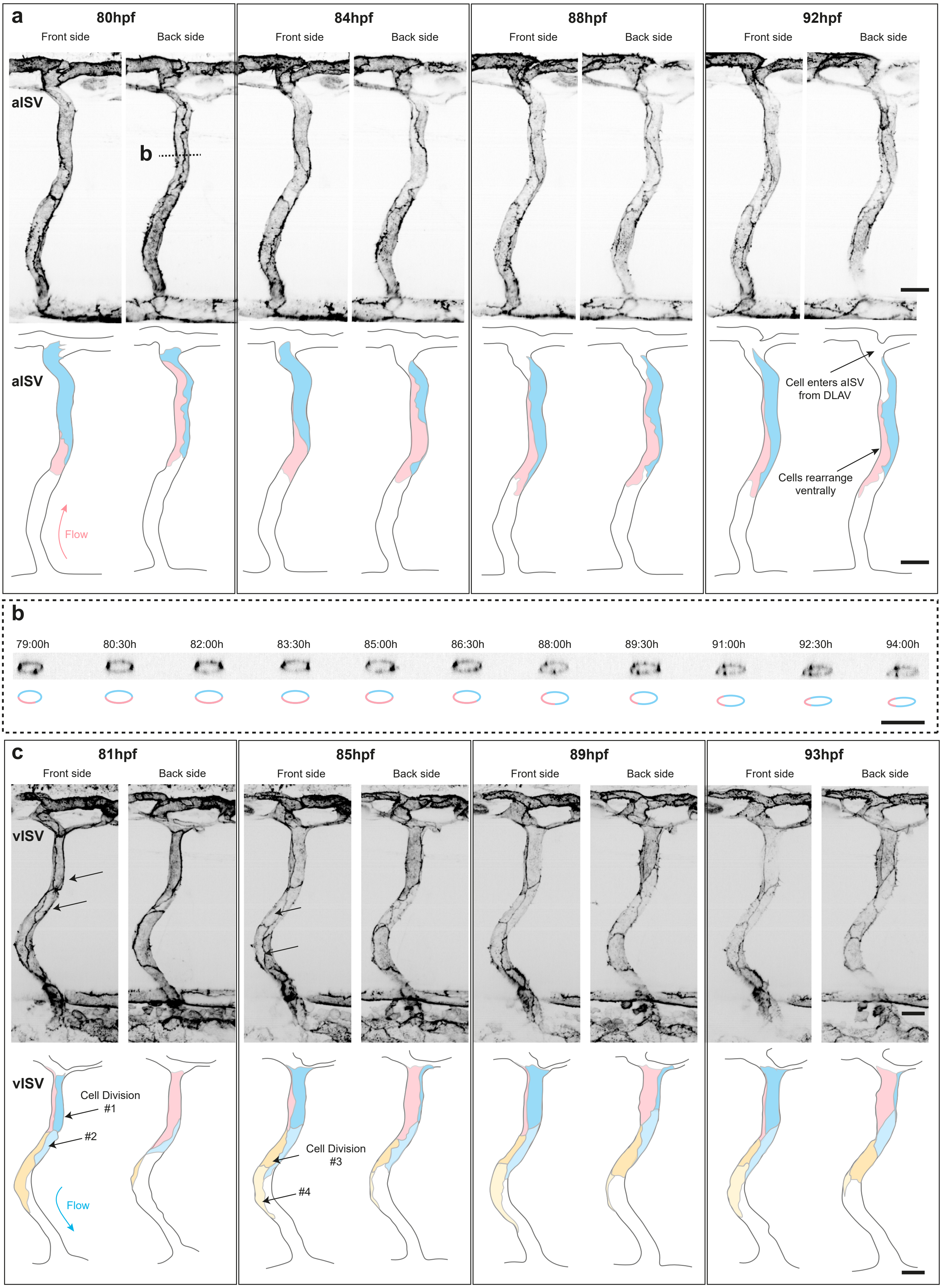

Supplementary Figure 3 Distinct cellular behaviours in aISVs and vISVs during vessel remodelling.
a, b, Still images of an aISV at 3-4 dpf (Supplementary Movie 2) showing cortical and junctional actin in Tg(fli1:GAL4FF)^{ubs3}; Tg(UAS:EGFP-UCHD)^{ubs18} embryos. Vessels are separated into front (left) and back (right) sides. Schematics highlight constituent cells. Cell rearrangements were observed in 17 movies. b Insets shows magnified region from a; coronal views correspond to the z-plane indicated by black serrated line in a. Scale bars, 20 μm (a) and 5 μm (b).
c Still images of a vISVs at 3-4 dpf (Supplementary Movie 4) showing cortical and junctional actin. Black arrows indicate cell divisions; daughter cells (#1-4) are marked in dark and light colours. Cell proliferation was frequently observed (in 16 movies).
Scale bar, 20 μm. ISV, intersegmental vessel; aISVs, arterial ISV; vISVs, venous ISV.

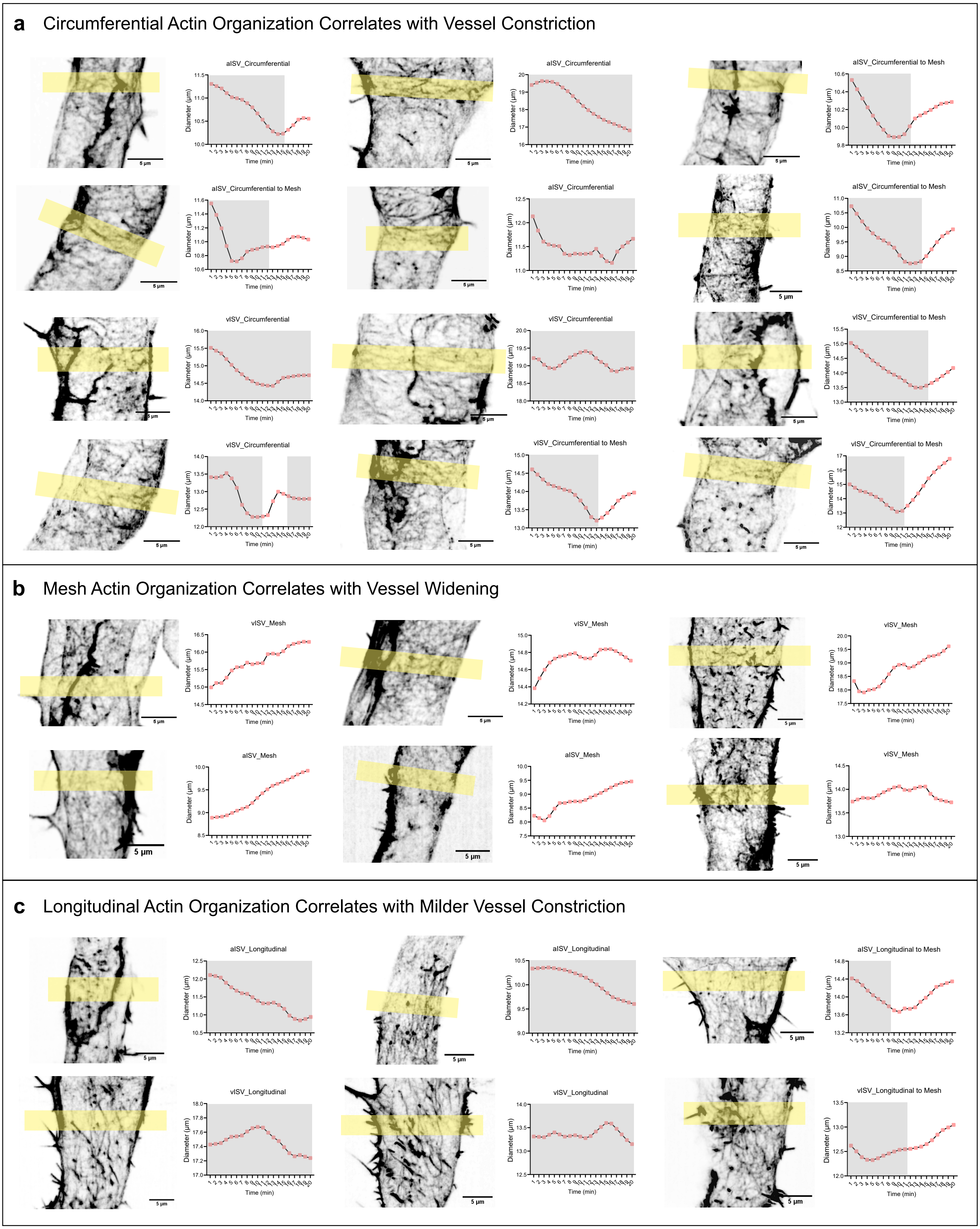

Supplementary Figure 4 Correlation between actin organization and vessel diameter dynamics.
a-c, ISV timelapse movies (left) with corresponding kymographs from the yellow ROI and vessel diameter measurements (right) at 2 dpf.
a Circumferential actin correlates with vessel constriction (12 movies: 6 aISVs, 6 vISVs). Grey rectangles indicate time periods when circumferential actin appears.
b Mesh actin correlates with vessel widening (6 movies: 2 aISVs, 4 vISVs).
c Longitudinal actin correlates with milder constriction (6 movies: 3 aISVs, 3 vISVs). In total 12 embryos used from 4 experiments. Grey rectangles indicate time periods when longitudinal actin appears. Scale bar, 5 µm. ISV, intersegmental vessel; aISVs, arterial ISV; vISVs, venous ISV.

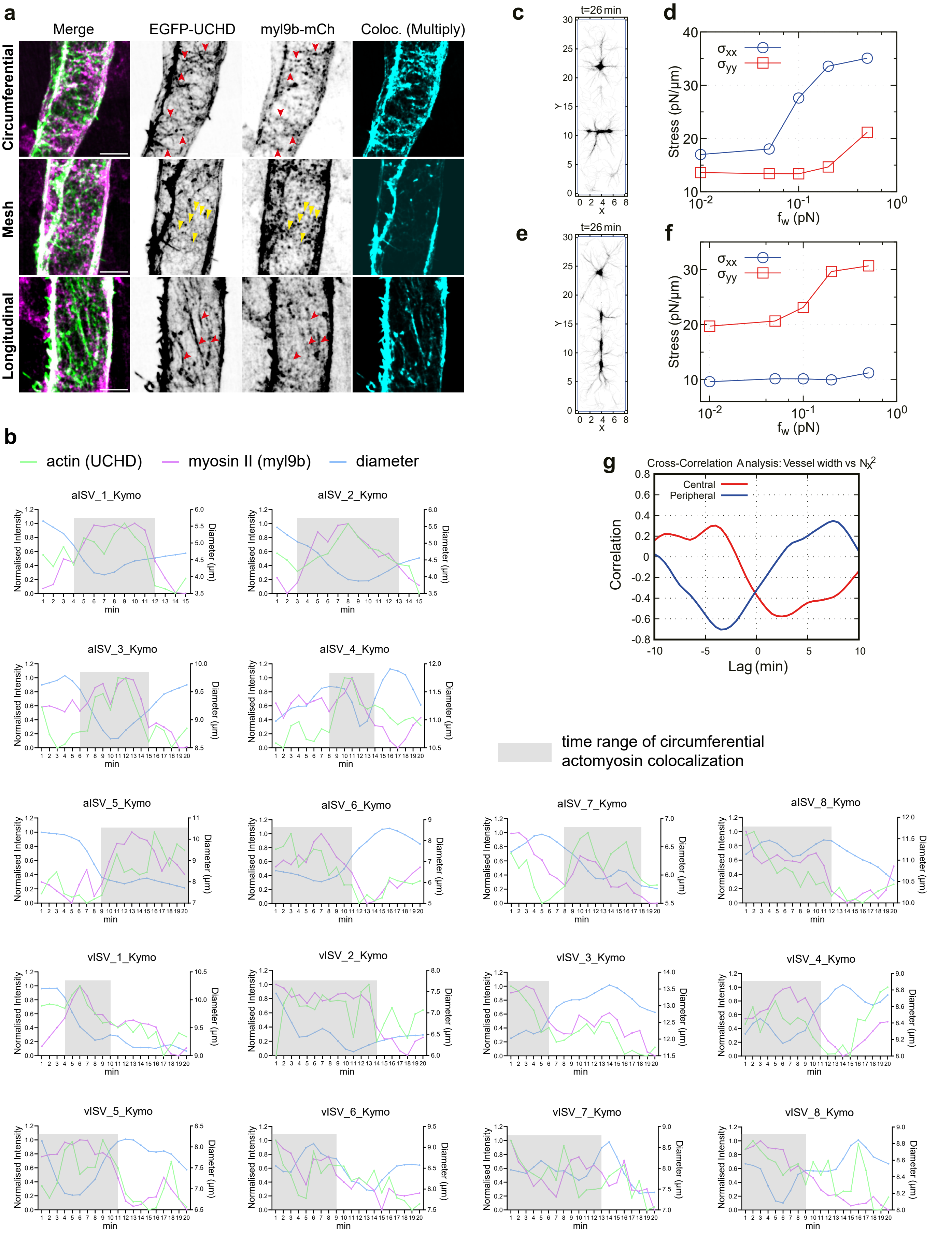

Supplementary Figure 5 Colocalization of actin and myosin II and mathematical simulation of vessel constriction.

a Representative images of circumferential, mesh, and longitudinal actin organizations (EGFP-UCHD) showing colocalization with non-muscle myosin II (myl9b-mCherry) at 2 dpf. Colocalization (cyan) was generated by multiplying the UCHD and mCherry channels in Fiji. Red arrowheads mark regions of actin-myosin II colocalization, and yellow arrowheads indicate myosin II localized within gaps of the mesh actin network.

b Quantification from time-lapse movies showing normalized $\log_{10}$ fluorescence intensity of UCHD and myl9b together with vessel diameter over time. Grey rectangles indicate periods when linear actomyosin colocalization appears. Each plot represents one ISV (n=16 plots: 8 aISVs and 8 vISVs movies, from 6 embryos, 3 experiments). ISV, intersegmental vessel; aISVs, arterial ISV; vISVs, venous ISV.

c, **e** Circumferential (c) and longitudinal (e) bundle formations in simulations of fixed boundary with a few actin filaments anchored along the longitudinal boundary (c) and midline (e), respectively. **d**, **f** Dependence of circumferential (σ_{xx}) and longitudinal (σ_{yy}) stress components on motor walking force f_w when circumferential (d) and longitudinal (f) bundles were formed.

g Cross correlation function between the vessel width and the circumferential alignment (N_x^2) for central (red) and the peripheral region (blue). The cross correlation function is $C(\tau)=\{(W(t+\tau) - \{W\} \cdot (N_x^2(t) - \{N_x^2\})\}_t / (\delta_W \delta_{N_x^2})$, where W is the vessel width and δ is the standard deviation.

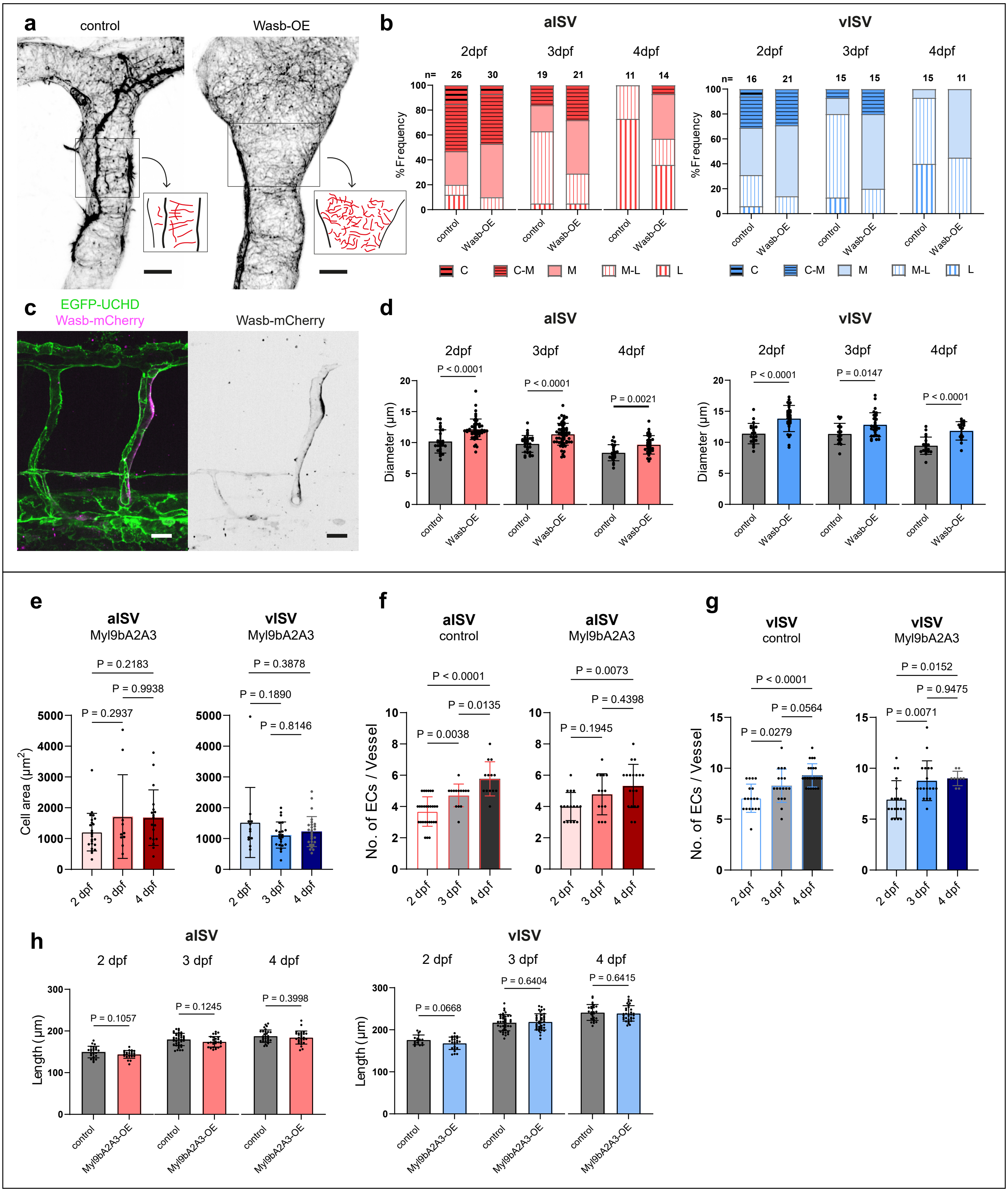

Supplementary Figure 6 Effects of Wasb and Myl9bA2A3 overexpression on actin organization and vessel morphology.

a, c Representative images of aISVs from 2 dpf Tg(fli1:GAL4FF)ubs3; Tg(UAS:EGFP-UCHD)ubs18 embryos with or without Wasb-mCherry overexpression (internal negative control). Actin organizations are highlighted in red in schematics. Scale bar, 10 µm (a) and 20 µm (c).

b Actin organization in control and Wasb-OE vessels from 2-4 dpf. Total number of ISVs (from 25/18/20 embryos at 2/3/4 dpf, 4/4/3 independent experiments) is indicated on the top of the bar. Fisher's exact test (M vs other AOs, control vs Wasb-OE): aISVs, p = 0.26/0.1861/0.04; vISVs, p=0.32/0.02/0.02 at 2/3/4 dpf.

d Quantification of vessel diameter from 2-4 dpf in control and Wasb-OE aISVs (n = 7/3/7 vs 14/7/14) and vISVs (n = 6/3/7 vs 6/7/11; from 10/6/16 embryos, 2/2/3 experiments). Each point represents one ISV. Data are mean ± SD; unpaired two-tailed t-test.

e Cell area of endothelial cells in control and Myl9bA2A3-OE vessels at 2-4 dpf (sample size as in Fig. 6h-i). Statistical significance was assessed by one-way ANOVA with Tukey's multiple comparisons test. Data are shown as mean ± SD.

f, g, Number of endothelial cells per vessel in control and Myl9bA2A3-OE aISVs (f) (n = 28/13/13 vs 18/14/19) and vISVs (g) (n = 18/18/23 vs 19/18/9) across 2-4 dpf (from 7/7/10 embryos at 2/3/4 dpf, 2/2/2 experiments). Statistical significance was assessed by one-way ANOVA with Tukey's multiple comparisons test. Data are mean ± SD.

h Vessel length of aISVs and vISVs in control and Myl9bA2A3-OE embryos from 2-4 dpf (sample size as in Fig. 6h-i). Each point represents one ISV. Data are mean ± SD; unpaired two-tailed t-test. ISV, intersegmental vessel; aISVs, arterial ISV; vISVs, venous ISV.

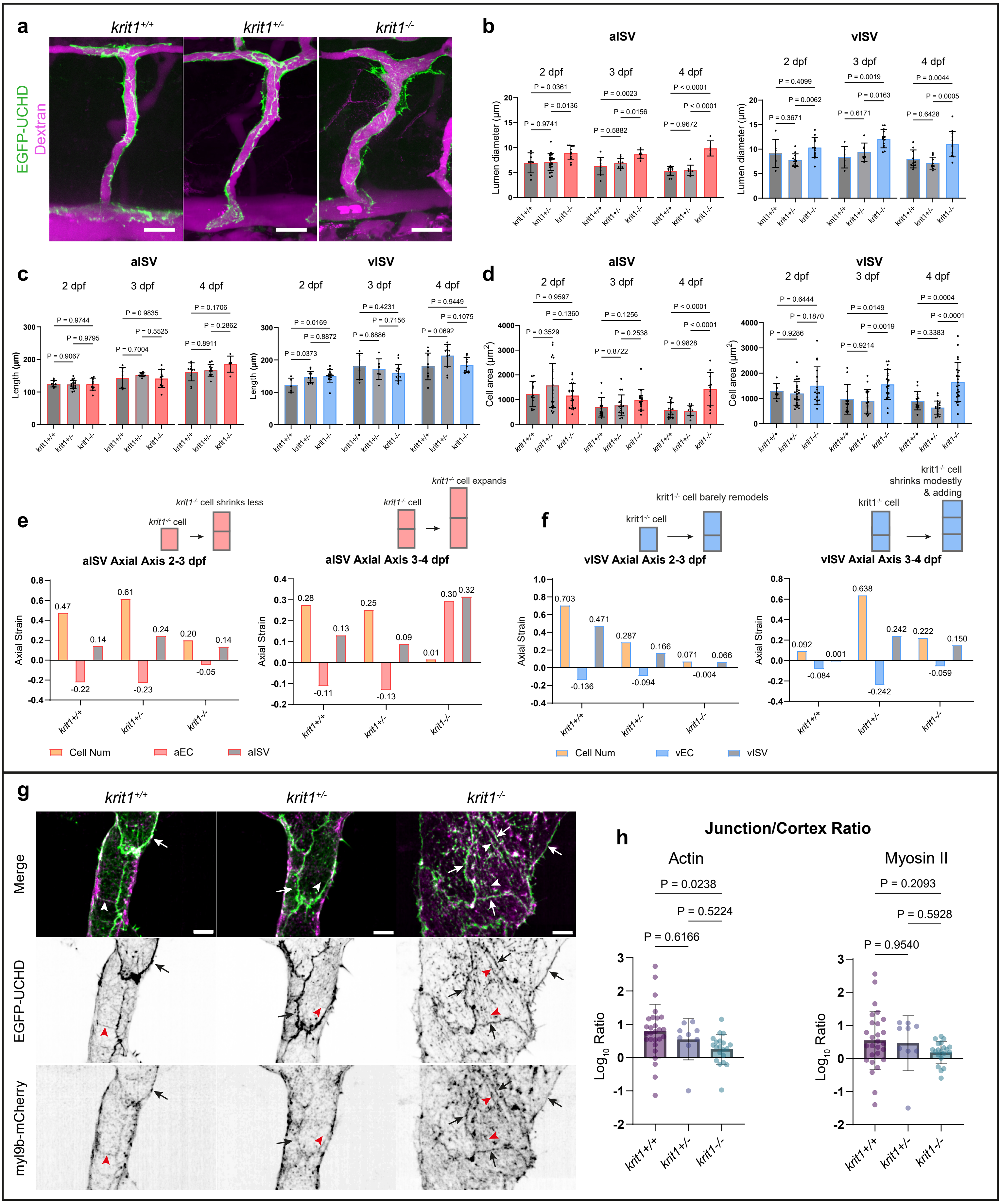

Supplementary Figure 7 Loss of Krit1 alters vessel morphology, endothelial strain, and actomyosin organization.

a Microangiography of transplanted aISVs containing *krit1*$^{+/+}$, *krit1*$^{+/-}$, or *krit1*$^{-/-}$ cells at 2 dpf. Dextran marks the vessel lumen. Scale bar, 20 μm.

b Quantification of lumen diameter in transplanted aISVs and vISVs containing *krit1*$^{+/+}$, *krit1*$^{+/-}$, or *krit1*$^{-/-}$ cells from 2-4 dpf (sample sizes as in Fig. 7g-h). Each point represents one ISV. Data are mean ± SD; one-way ANOVA with Tukey's multiple-comparisons test.

c Vessel length in aISVs and vISVs in transplanted aISVs between *krit1*$^{+/+}$ (n=9/7/10 at 2/3/4 dpf), *krit1*$^{+/-}$ (n=19/9/12 at 2/3/4 dpf), and *krit1*$^{-/-}$ (n=11/9/5 at 2/3/4 dpf), and vISVs between *krit1*$^{+/+}$ (n=5/6/9 at 2/3/4 dpf), *krit1*$^{+/-}$ (n=14/7/13 at 2/3/4 dpf), and *krit1*$^{-/-}$ (n=14/14/11 at 2/3/4 dpf). Embryos used: *krit1*$^{+/+}$ (N = 6/8/11), *krit1*$^{+/-}$ (N = 9/8/9), or *krit1*$^{-/-}$ (N = 5/5/6), from 6/7/9 experiments at 2/3/4 dpf.

d Cell area of aISVs and vISVs containing *krit1*$^{+/+}$, *krit1*$^{+/-}$, or *krit1*$^{-/-}$ endothelial cells (sample sizes as in Fig. 7g-h).

e, **f**, Axial strain in aISVs (e) and vISVs (f) from 2-4 dpf comparing *krit1*$^{+/+}$, *krit1*$^{+/-}$, or *krit1*$^{-/-}$ vessels (vessel length samples from Supplementary Fig. 7c; cell length samples from Fig. 7g-h).

g Representative images of ISVs from *krit1*$^{+/+}$, *krit1*$^{+/-}$, or *krit1*$^{-/-}$ embryos from an in-cross of *krit1*$^{+/-}$; *Tg(fli1:GAL4FF)*^{ubs3}; *Tg(UAS:EGFP-UCHD)*^{ubs18}; *Tg(6xUAS:myl9b-mCherry)*^{rk32}, showing actin (EGFP-UCHD) and non-muscle myosin II (myl9b-mCherry) localization. Scale bar, 5 μm. White and black arrows indicate cell-cell junction; white and red arrowheads indicate cortical actin.

h Quantification of junction/cortex normalized log_{10} fluorescence intensity ratios for actin and myosin II (*krit1*$^{+/+}$: n=27, 6 embryos; *krit1*$^{+/-}$: n=9, 1 embryo; *krit1*$^{-/-}$: n=20, 2 embryos, from 1 experiment). Data are mean ± SD; one-way ANOVA with Tukey's multiple-comparisons test. ISV, intersegmental vessel; aISV, arterial ISV; vISV, venous ISV.

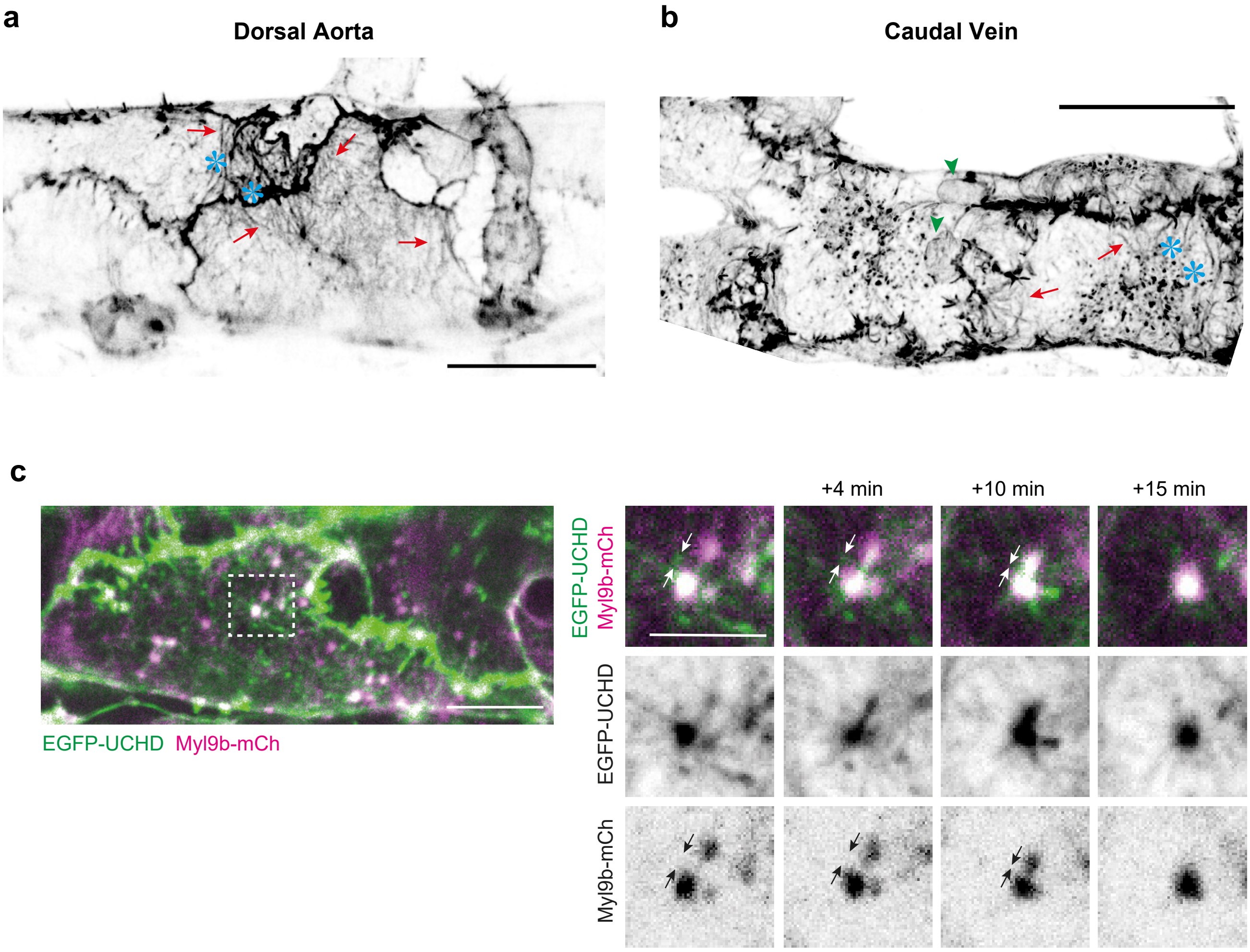

Supplementary Figure 8 Circumferential actin structures and actomyosin asters in major vessels.
a, b Representative images of circumferential actin organization in the dorsal aorta (DA) and caudal vein plexus (CVP) of *Tg(fli1:GAL4FF)*^{ubs3}; *Tg(UAS:EGFP-UCHD)*^{ubs18} embryos at 2 dpf. Red arrows indicate circumferential actin, blue asterisks indicate actin asters, and green arrowheads indicate bulbs. Scale bars, 20 µm.
c Representative images of actin asters (EGFP-UCHD) colocalizing with non-muscle myosin II (myl9b-mCherry) at 2 dpf. Insets from the white box show time-lapse sequence of converging asters over time. Scale bars, 10 µm (left) and 5 µm (right).

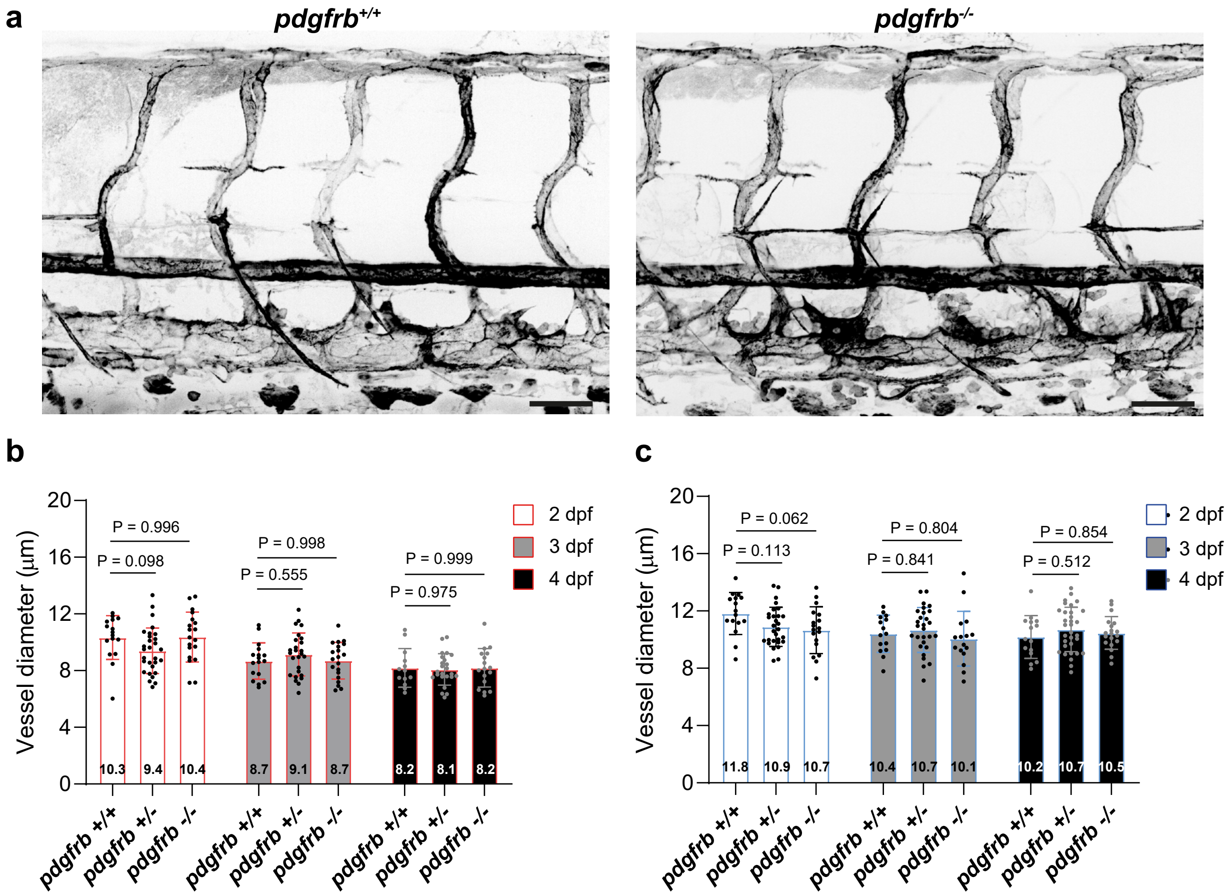

Supplementary Figure 9 No significant ISV diameter changes in pdgfrb mutants.

a Maximum intensity projections of confocal z-stacks showing ISVs in the zebrafish trunk of *pdgfrb*$^{+/-}$ and *pdgfrb*$^{-/-}$ embryos. *Tg(fli1:MYR-EGFP)*^{ncv2} label endothelial cytosol. Scale bar, 50 µm.

b,c, Quantification of vessel diameter in aISVs and vISVs from 2-4 dpf Vessel diameter in aISVs between *pdgfrb*$^{+/+}$ (n=16/17/13 at 2/3/4 dpf), *pdgfrb*$^{+/-}$ (n=29/26/25 at 2/3/4 dpf), and *pdgfrb*$^{-/-}$ (n=19/20/17 at 2/3/4 dpf), and vISVs between *pdgfrb*$^{+/+}$ (n=16/15/15 at 2/3/4 dpf), *pdgfrb*$^{+/-}$ (n=29/28/32 at 2/3/4 dpf), and *pdgfrb*$^{-/-}$ (n=18/17/19 at 2/3/4 dpf). Embryos used: *pdgfrb*$^{+/+}$ (N = 7/7/7), *pdgfrb*$^{+/-}$ (N = 14/14/14), or *pdgfrb*$^{-/-}$ (N = 9/9/9), from 2/2/2 experiments at 2/3/4 dpf. Each point represents one ISV. Data are mean ± SD; one-way ANOVA with Tukey's multiple-comparisons test.

ISV, intersegmental vessel; aISV, arterial ISV; vISV, venous ISV.

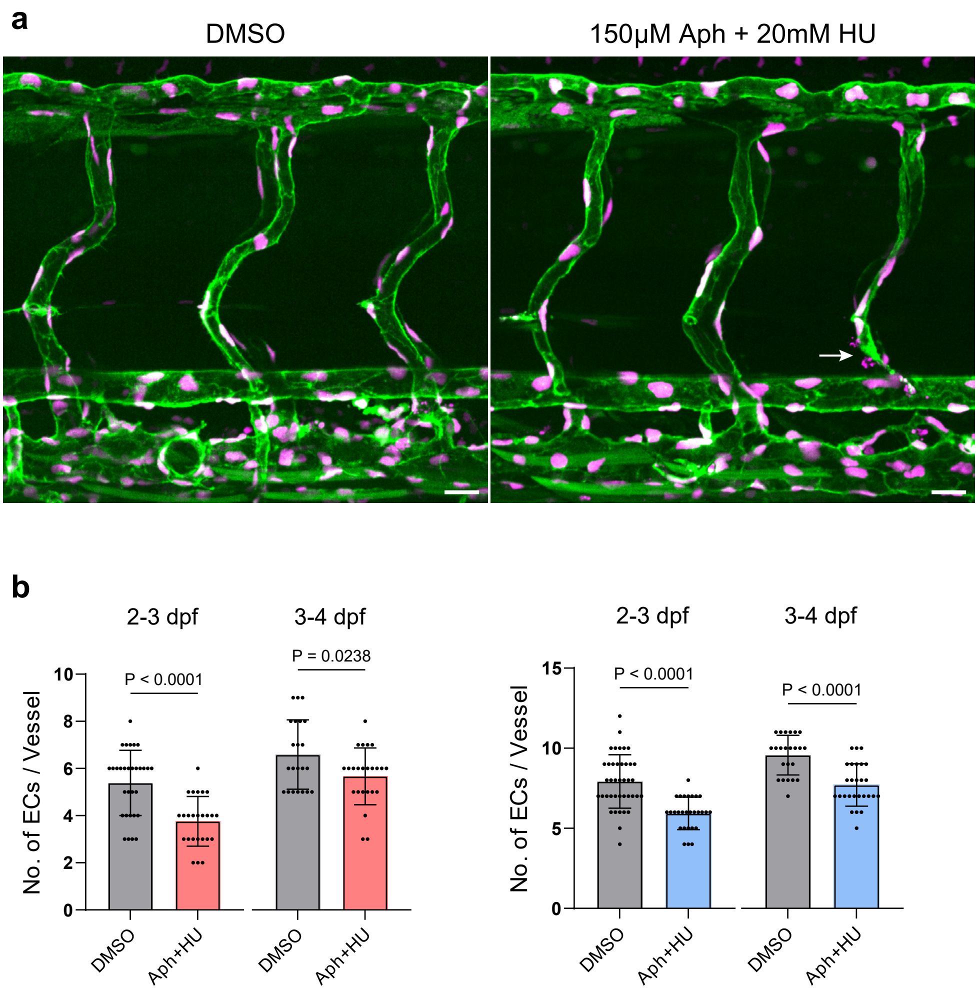

Supplementary Figure 10 Inhibition of cell proliferation decreases endothelial cell number per vessel.
a Maximum intensity projections of confocal z-stacks showing the same ISVs in the zebrafish trunk from 2-4 dpf. *Tg(fli1:h2bc1-mCherry)*^{ncv31}; *Tg(fli1ep:Lifeact-EGFP)*^{zf495} label endothelial nuclei and actin, respectively. White arrows indicate fragments from dying cells. Scale bar, 20 μm.
b, c Endothelial cell number per vessel in control and drug-treated embryos (150 μM aphidicolin + 20 mM hydroxyurea) from 2-3 and 3-4 dpf. aISVs: n = 31/22 control vs 25/24 treated at 2-3/3-4 dpf; vISVs: n = 40/23 control vs 28/27 treated at 2-3/3-4 dpf; DMSO embryos: N = 7/5; drug-treated embryos: N = 6/5 (1/1 experiment at 2-3/3-4 dpf). Each point represents one ISV. Data are mean ± SD; unpaired two-tailed t-test.

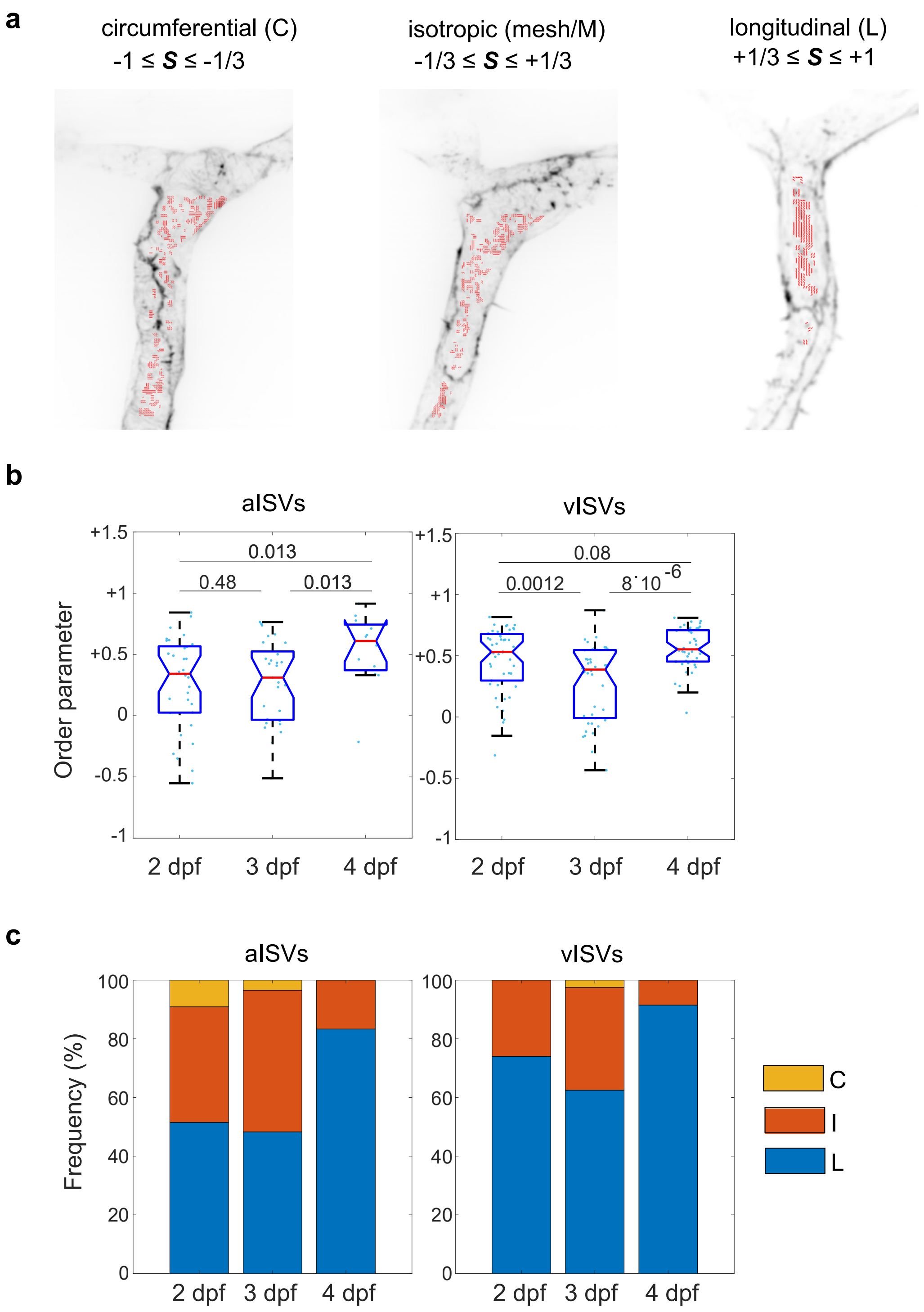

Supplementary Figure 11 Nematic order analysis of actin orientation in arterial and venous ISVs.

a Representative images of ISVs overlaid with nematic directors (red lines), indicating actin filament orientations. Predominant circumferential, isotropic, and longitudinal distributions of fibers were determined by calculating the median order parameter, $S=\langle\cos(2\theta)\rangle$, with θ is the angle between the nematic directors and the vessel orientation (see Supplementary Note 2). For the images shown, the order parameters are S = -0.45, 0.025, and 0.7 for circumferential, isotropic, and longitudinal, respectively.

b Distribution of the order parameters for aISVs and vISVs. Each data point in each box plot represents one analyzed vessel, with a total of $N_{2dpf} = 33$, $N_{3dpf} = 28$, $N_{4dpf} = 12$ for aISVs and $N_{2dpf} = 50$, $N_{3dpf} = 40$, $N_{4dpf} = 47$ for vISVs. Each box shows the median (red line), 25th and 75th percentiles (box), maximum and minimum without outliers (whiskers), and 95% confidence interval of the median (notches). P-values were calculated from Dunn's test of multiple comparisons after a significant Kruskal-Wallis test.

c Frequency plot showing the predominant presence of circumferential, isotropic, and longitudinal actin fiber orientations. The data are based on the distributions shown in b and are sorted as indicated in a.

Supplementary Note 1

Circumferential actomyosin bundles anchored by CCM1 drive endothelial cell contraction and vessel constriction

Yan Chen,[1] Nuria Taberner,[1] Jason da Silva,[1,2] Vivek Semwal,[1] Biplab Bhattacharjee,[1] Julia Eckert,[2] Igor Kondrychyn,[1] Mingzhao Hu,[1] Nitish Aswani,[1] Guihua Chen,[1] Yasushi Okada,[1,3] Anne Karine Lagendijk,[2,4] Tatsuo Shibata,[1] Satoru Okuda,[5] and Li-Kun Phng[1,*]

[1]*RIKEN Center for Biosystems Dynamics Research, Kobe, Japan.*
[2]*Institute for Molecular Bioscience, The University of Queensland, Brisbane, Queensland, Australia.*
[3]*Department of Cell Biology, Graduate School of Medicine, the University of Tokyo, Japan; Department of Physics, Graduate School of Science, the University of Tokyo, Japan; Universal Biology Institute (UBI), the University of Tokyo, Japan; International Research Center for Neurointelligence (WPI-IRCN), Institutes for Advanced Study, the University of Tokyo, Japan.*
[4]*School of Biomedical Sciences, Faculty of Medicine, The University of Queensland, Brisbane, Queensland, Australia.*
[5]*Nano Life Science Institute, Kanazawa University, Kanazawa, Japan.*

Model of cytoskeletal dynamics in endothelial cells

We consider a model of cytoskeleton dynamics in endothelial cells comprising filaments, motors, crosslinkers, and cell membranes with processes such as motor active force generation, polymerization and depolymerisation of filaments. Since the endothelial cells that surround the blood vessel are flat cells, we approximately describe the system by a two dimensional model. We also considered turnover of filaments and crosslinkers to maintain the dynamic property of the system. To facilitate the circumferential bundle formation, we introduced anchored filaments at the vertical (longitudinal) boundary. The model used in this study constitutes an extension of our previously introduced model [1]. Below, we present the detail of the model.

a. Actin filaments Actin filaments are represented as semi-flexible polymers, modeled as chains of beads connected by springs with rest length l_0^f. The filament lengths are dynamic, governed by the rates of polymerization r^f_{pol} and depolymerization r^f_{dpol} at the filament ends. The system contains a total of N_f filaments.

The equation of motion for the position $\mathbf{r}_{(f,i)}$ of bead i of filament f is given by

$$\gamma^f \dot{\mathbf{r}}_{(f,i)} = \mathbf{F}^{\text{m}}_{(f,i)} + \mathbf{F}^{\text{str}}_{(f,i)} + \mathbf{F}^{\text{bend}}_{(f,i)}, + \sum_j \mathbf{F}^{\text{rep}}_{(f,i)\leftarrow j} + \boldsymbol{\zeta}_{(f,i)}. \tag{1}$$

The first term on the right hand side is the force from attached motors. The motor force due to head h of motor m is given by

$$\mathbf{F}^{\text{m}}_{(f,i)} = \sum_{m,h} \alpha_{(m,h)} f^h_m + \sum_{m,h} (1-\alpha_{(m,h)}) f^h_m, \tag{2}$$

where the first and second summation are taken for segments between $\mathbf{r}_{(f,i-1)}$ and $\mathbf{r}_{(f,i)}$, and between $\mathbf{r}_{(f,i)}$ and $\mathbf{r}_{(f,i+1)}$, respectively. The terms $\mathbf{F}^{\text{str}}_{(f,i)}$ and $\mathbf{F}^{\text{bend}}_{(f,i)}$ represent stretching and bending elasticity, respectively, $\mathbf{F}^{\text{rep}}_{(f,i)\leftarrow j}$ denotes repulsion from membrane bead j, and $\boldsymbol{\zeta}_{(f,i)} = (\zeta_{(f,i),x}, \zeta_{(f,i),y})$ is Gaussian noise with $\langle \zeta_{(f,i),j} \rangle = 0$ and $\langle \zeta_{(f,i),j}(t) \zeta_{(f',i'),j'}(t') \rangle = 2\gamma^f k_B T \delta_{ff'} \delta_{ii'} \delta_{jj'} \delta(t-t')$.

b. Myosin motors and crosslinkers Motors are represented as central particles m with two heads ($h = 1, 2$). The positions of the central particle and heads are given by $\mathbf{r}_m$ and $\mathbf{r}_{(m,h)}$, respectively. The tethered heads may attach to the segment between $\mathbf{r}_{(f,i)}$ and $\mathbf{r}_{(f,i+1)}$ of filament f, occupying a position

$$\mathbf{r}_{(m,h)} = \mathbf{r}_{(f,i)} + \alpha_{(m,h)}(\mathbf{r}_{(f,i+1)} - \mathbf{r}_{(f,i)}), \tag{3}$$

where $\alpha_{(m,h)} \in [0,1]$ is the fractional coordinate along the segment between $\mathbf{r}_{(f,i)}$ and $\mathbf{r}_{(f,i+1)}$.

In the filament-fixed frame, only the relative coordinate α evolves as

$$\gamma^m l_{(f,s)} \dot{\alpha}_{(m,h)} = f_w, \tag{4}$$

where f_w is the walking force driving the head towards the filament's plus end. For the crosslinkers, $f_w = 0$. The equation of motion for the motor's central particle is

$$\gamma^m \dot{\mathbf{r}}_m = \boldsymbol{\zeta}_m - \sum_{h=1}^{2} \mathbf{f}^h_m, \tag{5}$$

*Corresponding author: likun.phng@riken.jp

where $\boldsymbol{\zeta}_m = (\zeta_{m,x}, \zeta_{m,y})$ is Gaussian noise with $\langle \zeta_{m,i} \rangle = 0$ and $\langle \zeta_{m,i}(t)\zeta_{m',i'}(t') \rangle = 2\gamma^m k_B T \delta_{mm'}\delta_{ii'}\delta(t-t')$, and

$$\mathbf{f}^h_m = \mathbf{F}^{\rm str}_{m,h} + \mathbf{F}^{\rm bend}_{m,h} \tag{6}$$

is the total force on motor head h from stretching and bending, summed over the two linked filaments.

c. Cell membrane To mimic a flat endothelial cell, we consider a rectangular area of 8 μm× 30μm enclosed by cell membrane. The cell membrane consists of consecutive beads. To study whether the circumferential bundle can be induced by the anchored filaments (see below), we first considered a situation where the position of the beads were fixed. We then introduced a deformable boundary consisting of consecutive beads connected by springs, with stretching and bending elasticity, into the vertical (longitudinal) boundary to see whether the contractile force can reduce the width of the cell. The position of a membrane bead i is denoted by $\mathbf{r}^b_i$. The temporal evolution of this position is given by

$$\gamma^b \dot{\mathbf{r}}^b_i = \sum_j \mathbf{F}^{\rm rep}_{s\leftarrow j} + \mathbf{F}^{\rm str}_i + \mathbf{F}^{\rm bend}_i + \boldsymbol{\zeta}_i, \tag{7}$$

where $\mathbf{F}^{\rm rep}_{s\leftarrow j}$ is the repulsive force on the membrane bead from nearby filament bead j, and $\mathbf{F}^{\rm str}_i$ and $\mathbf{F}^{\rm bend}_i$ are the stretching and bending forces due to the membrane's elasticity. $\boldsymbol{\zeta}_i = (\zeta_{i,x}, \zeta_{i,y})$ is Gaussian noise with $\langle \zeta_{i,j} \rangle = 0$ and $\langle \zeta_{i,j}(t)\zeta_{i',j'}(t') \rangle = 2\gamma^b k_B T \delta_{ii'}\delta_{jj'}\delta(t-t')$.

Here, the stretching and bending forces are given by

$$\mathbf{F}^{\rm str}_{(i)} = -\frac{\partial}{\partial \mathbf{r}_{(i)}} \sum_{j\in i\pm 1} U^{\rm str}(r_{ij}) \tag{8}$$

$$\mathbf{F}^{\rm bend}_{(i)} = -\frac{\partial}{\partial \mathbf{r}_{(i)}} U^{\rm bend}_{(i)}(\mathbf{r}_{(i-1)i}, \mathbf{r}_{i(i+1)}), \tag{9}$$

with

$$U^{\rm str}(r_{ij}) = \frac{1}{2}k^{\rm str}\left(r_{ij} - l_0\right)^2 \tag{10}$$

$$U^{\rm bend}_{(i)} = -k^{\rm bend}\hat{\mathbf{r}}_{(s-1)s}\cdot\hat{\mathbf{r}}_{s(s+1)} \tag{11}$$

where $k^{\rm str}$ and $k^{\rm bend}$ are the elastic modulus and the bending rigidity, respectively, $\mathbf{r}_{ij} = \mathbf{r}_j - \mathbf{r}_i$, $r = |\mathbf{r}|$, and and $\hat{\mathbf{r}} = \mathbf{r}/r$. The repulsion force between two beads i and j of the system is given by

$$\mathbf{F}^{\rm rep}_{i\leftarrow j} = -\frac{\partial}{\partial \mathbf{r}_i} U^{\rm rep}(r_{ij}), \tag{12}$$

with

$$U^{\rm rep}(r_{ij}) = \begin{cases} \dfrac{\epsilon}{\left(1 - \frac{\sigma^{\rm rep}}{r_{\rm cut}}\right)}\left[\left(\dfrac{\sigma^{\rm rep}}{r_{ij}}\right) - \left(\dfrac{\sigma^{\rm rep}}{r_{\rm cut}}\right)\right], & (r_{ij} < r_{\rm cut}), \\ 0 \quad (\text{otherwise}), \end{cases} \tag{13}$$

where ϵ, $\sigma^{\rm rep}$ and $r_{\rm cut}$ are the strength, the decay length and the range of the repulsion force, respectively.

d. Anchored filaments To facilitate the bundle formation in the circumferential direction, we introduced anchored filaments at the vertical (longitudinal) boundary. The plus end of each tag filament is anchored to the boundary. These filaments have the same length as non-anchored filaments, but are excluded from polymerization and depolymerization dynamics.

e. Filament Turnover Following the complete dynamical evolution, filament turnover is introduced for non-tag filaments. Filaments stochastically dissociate from the system and reappear at random locations at a characteristic turnover rate, modeling the dynamic remodeling of the cytoskeletal network.

f. Crosslinker Turnover Crosslinkers stochastically detach from filaments, reflecting the finite lifetime of crosslinker-filament interactions influenced by thermal fluctuations. Unbound-crosslinkers attach new positions immediately based on the density dependent binding probability described below.

g. Motor and Crosslinker Re-attachment To avoid too much accumulation of motors and crosslinkers to particular filaments, we introduced a density dependent binding probability. The probability of attaching to segment i of a filament is

$$p_i = \frac{\exp(-\beta n_i)}{\sum_{j=1}^{N}\exp(-\beta n_j)}, \tag{14}$$

where n_i is the number of bound motors and crosslinkers at segment i, β is a constant, and N is the total number of segments in the system. In the current simulation $\beta = 1$.

[1] Tarama, M. and Shibata, T. Pattern formation and the mechanics of a motor-driven filamentous system confined by rigid membranes. *Phys. Rev. Res.* 4, 043071 (2022).

[2] Fang, X., Kruse, K., Lu, T. and Wang, J. Nonequilibrium physics in biology. *Rev. Mod. Phys.* **91**, 045004 (2019).

[3] Gittes, F., Mickey, B., Nettleton, J. and Howard, J. Flexural rigidity of microtubules and actin filaments measured from thermal fluctuations in shape. *J. Cell Biol.* **120**, 923–934 (1993).

[4] Rahmani, H., Ma, W., Hu, Z., Daneshparvar, N., Taylor, D. W., McCammon, J. A., Irving, T. C., Edwards, R. J. and Taylor, K. A. The myosin II coiled-coil domain atomic

structure in its native environment. *Proc. Natl. Acad. Sci. USA* **118**, e2024151118 (2021).
[5] Oldfors, A. Hereditary myosin myopathies. *Neuromuscul. Disord.* **17**, 355–367 (2007).
[6] Erdmann, T. and Schwarz, U. S. Stochastic force generation by small ensembles of myosin II motors. *Phys. Rev. Lett.* **108**, 188101 (2012).

**Parameters**	Symbols	Observed values	In Silico	References
Energy scale	$k_B T$	$4.12 \times 10^{-3} pN\mu m$	$4.12 \times 10^{-3} pN\mu m$	
Cytosolic viscosity	η_c	1 Pa s	1 Pa s	[2]
**Actin**				
Number of Actin Filaments	N_f		1500	
Number of Tag Actin Filament	$N_{f,tag}$		40	
Number of Tag			4	
Rest segment length	l_0^f		$0.1\mu m$	
Bending rigidity	k_f^{bend}		$0.2 pN\mu\ m$	
Persistence length	$l_f^p = \frac{k_f l_0^f}{k_B T}$		$24.1\mu m$	[3]
Full length	l_f	$\sim$ few μm	$2\mu m$	[4]
Diameter	σ^f	$0.009\mu m$	0.01 µm	[3]
Cytosolic friction	$\gamma^f = 3\pi\eta_c(3\sigma^f + 2l_0^f)/5$		0.09 pN sec µm^{-1}	
Repulsion strength	ϵ		$10 pN\ \mu m$	
Repulsion depth	σ^{rep}		$0.05\mu m$	
Repulsion cut-off length	r_{cut}		$0.21\mu m$	
Stretching modulus	k_f^{str}		$10 pN$	
**Myosin Motor**				
Number of Myosin Motor	N_m		2500	
Rest length segment	l_0^m		$0.05\mu m$	
Total length	l_m		$0.10\mu m$	[4]
Diameter	σ^m	$0.01 - 0.03\mu m$	$0.01\mu m$	[5]
Cytosolic friction	$\gamma^m = 6\pi\eta_c(3\sigma^m + 2l_0^m)/5$		0.04 pN sec µm^{-1}	
Motor walking force	f_w		0.20 pN	[6]
Stretching modulus	k_m^{str}		10 pN	
Bending rigidity	k_m^{bend}		0.10pN µm	
**Crosslinker**				
Number of Crosslinker			6000	
Total length	l_{cr}		0.1 µm	[5]
Rest length segment	l_0^{cr}		0.05 µm	
Diameter	σ^{cr}		0.01 µm	
Cytosolic friction	$\gamma^{cr} = 6\pi\eta_c(3\sigma^{cr} + 2l_0^{cr})/5$		0.04pN sec µm^{-1}	
Crosslinker walking force			0.0pN	
Stretching modulus	k_{cr}^{str}		10 pN	
Bending rigidity	k_{cr}^{bend}		0.57pN µm	
Turnover rate of a filament	r_{turn}^f		0.001 sec^{-1}	
Turnover rate of a Crosslinker	r_{turn}^{cr}		0.004 sec^{-1}	
Polymerisation rate a filament	r_{pol}^f		0.002 sec^{-1}	
Depolymerisation rate of a filament	r_{dpol}^f		0.002 sec^{-1}	

Supplementary Table 1: Parameters values used in the simulation.

Supplementary Note 2

Order parameter of actin filaments

The order parameter analysis was performed using MATLAB R2021b.

First, the images were pre-processed to remove the background, i.e., the signal outside of the vessel, and the junctional signal to analyse the actin filaments. The removal of the background signal was achieved by creating a black-and-white mask of the vessel, with '0' representing black and '1' representing white. This was done by (i) applying a Gaussian filter with a kernel of standard deviation 5 to smooth the intensity signal, and (ii) setting intensity pixels with values below the image mean to '0'. The largest connected area of '1's was considered the mask for the vessel. Subsequently, the lower and upper parts of the vessel were manually removed from the mask, leaving a mask of a unidirectional vessel. The intensity threshold used to remove the junction was set manually for all images. This junction mask was then subtracted from the full vessel mask to obtain the final mask used for actin filament orientation analysis within the region of interest.

The orientation analysis of the actin filaments was performed using the ImageJ plugin OrientationJ implemented in MATLAB [1]. OrientationJ computes the eigendirection of the structure tensor for each pixel, using a Gaussian-shaped window with $\sigma = 3$, as well as the local coherency. To focus on coherent structures, here the actin filaments, pixels with coherency < 0.8 were excluded. The orientation of each pixel, n, at position r_n was converted into a complex order parameter, $\varphi(r_n)$, and the average was taken over N pixels within a window size of 10 x 10 pixels:

$$\psi(r_w) = \sum_{n=0}^{N} \varphi(r_n) = \sum_{n=0}^{N} e^{i2\alpha_n} , \quad (15)$$

where α is the angle of the local orientation with respect to an arbitrary axis obtained by OrientationJ. The phase of the complex order parameter at position, r_w, with grid distance, W = 10 pixels, is then given by

$$\vartheta(r_w) = \frac{\mathrm{Arg}(\psi(r_w))}{2}. \quad (16)$$

The nematic director was calculated as

$$n(r_w) = (\cos(\vartheta(r_w)), -\sin(\vartheta(r_w))). \quad (17)$$

To identify the orientation of the nematic director relative to the vessel orientation, the vessel mask was reduced to a single-pixel-wide line, i.e., a center line and tangents along this line were obtained. These tangents at positions, r_c, with distance of 50 pixels along the center line, were calculated by selecting pixels within a radius of 50 pixels and fitting a linear regression by singular value decomposition. The angle between $n(r_w)$ and the nearest tangent, $t(r_c)$, was then calculated as

$$\theta(r_w) = \mathrm{acos}(\frac{n(r_w) \cdot t(r_c)}{|n(r_w)||t(r_c)|}), \quad (18)$$

The median order parameter for each vessel was computed as

$$S =< \cos(2\theta) >, \quad (19)$$

ranging between -1 and 1, where -1 represents perfect circumferential alignment, 0 indicates isotropic orientation, and 1 corresponds to perfect longitudinal alignment. Values outside the mask were excluded from the analysis. For the analysis, we sorted mean order parameters as follows: circumferential: $-1 \leq S \leq -1/3$; isotropic, e.g. mesh: $-1/3 < S \leq +1/3$; longitudinal: $1/3 < S \leq 1$. Vessels with less than 180 nematic directors, corresponding to approximately 10 % of the maximum amount, have not been considered for the analysis.

References

1. Sage, D. MIJ: Running ImageJ and Fiji within Matlab. *MATLAB Central File Exchange*. (2025)
