# Supplementary material for: Circumferential actomyosin bundles anchored by CCM1 drive endothelial cell contraction and vessel constriction
Source: Nat Commun. 2025 Dec 27;17:1056. doi: 10.1038/s41467-025-67820-3 (PMC12848307; doi:10.1038/s41467-025-67820-3)
Supplement: Supplementary file 2 — Description of Additional Supplementary File [file 41467_2025_67820_MOESM2_ESM.pdf]

Description of Additional Supplementary Files

Supplementary Data-
Supplementary Data 1: Plasmid information (Excel file).

Supplementary Movies-

- **Supplementary Movie 1**: Cell rearrangement and self-seam junction formation in aISVs at 2–3 dpf (related to Fig. 3a).
- **Supplementary Movie 2:** Cell rearrangement in aISVs at 3–4 dpf (related to Supplementary Fig. 3a).
- **Supplementary Movie 3**: Cell rearrangement and division in vISVs at 2–3 dpf (related to Fig. 3c).
- **Supplementary Movie 4:** Cell rearrangement and division in vISVs at 3–4 dpf (related to Supplementary Fig. 3c).
- **Supplementary Movie 5:** Circumferential actin bundles anchored to the cell membrane in aISVs (related to Fig. 4c).
- **Supplementary Movie 6:** Circumferential actin bundles connected to cell–cell junctions in vISVs (related to Fig. 4d).
- **Supplementary Movie 7:** Circumferential actin bundles linking the cell membrane and cell–cell junctions in vISVs (related to Fig. 4d).
- **Supplementary Movie 8:** Colocalization of circumferential actin bundle and myosin II in aISVs (related to Fig. 5a).
- **Supplementary Movie 9**: 3D rendering of circumferential actomyosin bundle (related to Fig. 5b).
- **Supplementary Movie 10:** Simulation of circumferential bundle formation under fixed boundaries (related to Supplementary Fig. 5c).
- **Supplementary Movie 11:**Simulation of longitudinal bundle formation under fixed boundaries (related to Supplementary Fig. 5e).

- **Supplementary Movie 12:** Simulation of membrane deformation driven by circumferential bundles (related to Fig. 5d).
- **Supplementary Movie 13:** Laser ablation of circumferential actin in aISVs (related to Fig. 5h).
- **Supplementary Movie 14:** Laser ablation of mesh actin in aISVs (related to Fig. 5h).
- **Supplementary Movie 15: Laser ablation of longitudinal actin in vISVs (related to Fig. 5h).**
- **Supplementary Movie 16:** Circumferential actin bundles in the dorsal aorta (DA) (related to Supplementary Fig. 8a).
- **Supplementary Movie 17:** Circumferential actin bundles in the caudal vein plexus (CVP) (related to Supplementary Fig. 8b).
- **Supplementary Movie 18:** Converging actomyosin asters in the dorsal aorta (DA) (related to Supplementary Fig. 8b
