# Supplementary figures and images for: Circumferential actomyosin bundles anchored by CCM1 drive endothelial cell contraction and vessel constriction
Source: Nat Commun. 2025 Dec 27;17:1056. doi: 10.1038/s41467-025-67820-3 (PMC12848307; doi:10.1038/s41467-025-67820-3)

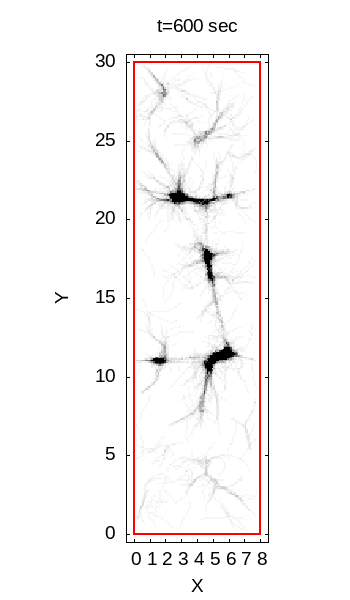

Supplement: Supplementary file 13 — Supplementary Movie 10 [file 41467_2025_67820_MOESM13_ESM.gif]

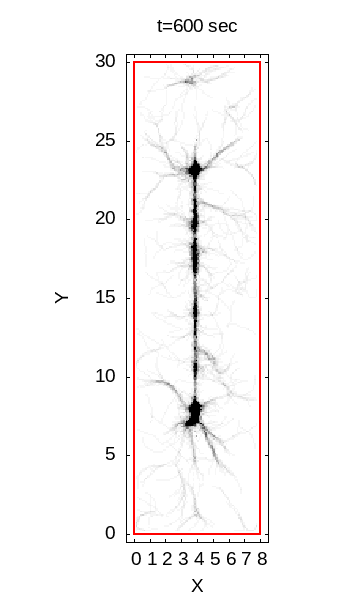

Supplement: Supplementary file 14 — Supplementary Movie 11 [file 41467_2025_67820_MOESM14_ESM.gif]

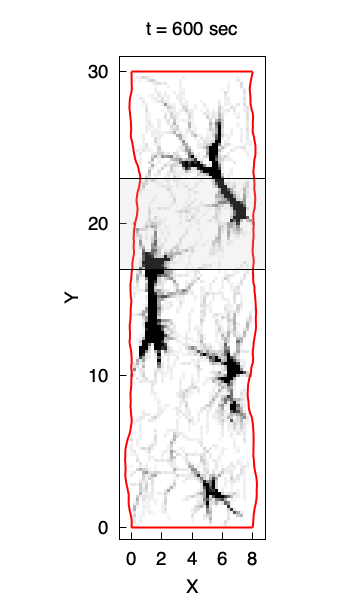

Supplement: Supplementary file 15 — Supplementary Movie 12 [file 41467_2025_67820_MOESM15_ESM.gif]
